# Factors influencing fall prevention programmes across three regions of the UK: the challenge of implementing and spreading the Falls Management Exercise (FaME) programme in a complex landscape

**DOI:** 10.1093/ageing/afaf083

**Published:** 2025-04-10

**Authors:** Jodi P Ventre, Fay Manning, Aseel Mahmoud, Grace Brough, Stephen Timmons, Helen Hawley-Hague, Dawn A Skelton, Victoria A Goodwin, Chris J Todd, Denise Kendrick, Pip Logan, Elizabeth Orton

**Affiliations:** National Institute for Health and Care Research, Applied Research Collaboration Greater Manchester, School of Health Sciences, Faculty of Biology, Medicine and Health, The University of Manchester, Manchester, UK; NIHR Applied Research Collaboration Southwest Peninsula, University of Exeter Medical School, Exeter, UK; NIHR Applied Research Collaboration Southwest Peninsula, University of Exeter Medical School, Exeter, UK; School of Medicine, University of Nottingham, Nottingham, UK; Business School, University of Nottingham, Nottingham, UK; National Institute for Health and Care Research, Applied Research Collaboration Greater Manchester, School of Health Sciences, Faculty of Biology, Medicine and Health, The University of Manchester, Manchester, UK; School of Physiotherapy and Paramedicine, Research Centre for Health (ReaCH), Glasgow Caledonian University, Glasgow, UK; NIHR Applied Research Collaboration Southwest Peninsula, University of Exeter Medical School, Exeter, UK; National Institute for Health and Care Research, Applied Research Collaboration Greater Manchester, School of Health Sciences, Faculty of Biology, Medicine and Health, The University of Manchester, Manchester, UK; Manchester Academic Health Science Centre Manchester, Manchester, UK; Manchester University NHS Foundation Trust, Manchester, UK; School of Medicine, University of Nottingham, Nottingham, UK; School of Medicine, University of Nottingham, Nottingham, UK; School of Medicine, University of Nottingham, Nottingham, UK

**Keywords:** fall prevention, implementation, the Falls Management Exercise (FaME), qualitative research, commissioning, older people

## Abstract

**Background:**

The occurrence of falls in adults 65+ years remains a common and costly issue worldwide. There is current evidence to suggest that falls can be prevented using evidence-based strength and balance interventions, such as the six-month Falls Management Exercise (FaME) programme. Perspectives of multiple key partners and providers of the FaME programme could inform future implementation and fall prevention strategies.

**Methods:**

Partners and providers involved in local community fall prevention pathways were purposefully recruited from three geographical areas across the UK. Semistructured interviews were conducted to gain a broad understanding of factors affecting the adoption, implementation and spread of FaME. Data were analysed using an inductive thematic approach and mapped to the Consolidated Framework for Implementation Research (CFIR).

**Results:**

Data from 25 participant interviews and document analysis revealed 11 themes organised within five CFIR domains—the innovation (3), outer setting (3), inner setting (1), characteristics of individuals (1) and process (2).

**Conclusion:**

The adoption, implementation and spread of FaME into community settings is complex and faces multiple health system challenges. For adoption and implementation to be facilitated, FaME programmes must demonstrate effectiveness and fit the local needs of those receiving the intervention. For spread to occur, influential decision-makers and funders must support wider programme rollout whilst also securing sufficient expert capacity to deliver the programme and ensure monitoring is in place to determine effectiveness of provision for older adults.

## Key Points

- Adoption, implementation and spread of the Falls Management Exercise (FaME) programme faces multiple challenges from within and outside of organisations.
- Adoption and implementation are reliant upon meeting the needs of localities and aligning with national guidelines.
- Support from influential decision-makers and funders is required to spread programmes such as the FaME.
- Spread can be limited by the availability of specialist Postural Stability Instructor workforce and is dependent on their knowledge.
- Translation of evidence into practice remains a time-consuming process that should be supported using an implementation strategy.

## Background

Globally, falls are a major public health concern. Falls are the second leading cause of unintentional injury-related deaths worldwide, with an estimated 684 000 fatal falls occurring each year [1]. In the United Kingdom (UK), the proportion of the population aged 65+ is projected to increase to 24% by 2038 [2] increasing the need for a greater understanding of ageing health challenges and related policy responses. Older adults who fall are known to present with increased concerns about falling [3, 4], depression [5], functional decline and overall greater dependence on caregivers [6, 7]. The wider societal impact is that of increased costs on health and social care systems [8] as a result of injurious falls. More than half of all falls that occur take place in independent living community settings [9, 10], highlighting the importance of evidence-based community fall prevention interventions.

The Falls Management Exercise (FaME) programme has been shown in two randomised controlled trials and a ‘real-world’ implementation study [11] to be effective at reducing the rate of falls, [12, 13], to increase moderate-intensity physical activity and confidence and reduce concerns about falling [14]. The FaME programme is a 24-week community-based multicomponent progressive exercise programme that is individualised and has embedded behavioural support [12]. The components of fitness training are similar to other fall prevention programmes (Otago [15]), however, FaME places greater emphasis on reactive and compensatory stepping, upper body and core strength and uniquely has a strong focus on falls management by retraining and maintaining the skill of getting up from the floor.

FaME is delivered patchily across the UK, despite being available as an exercise intervention to prevent falls since 2000. All classes are delivered by a specialist Postural Stability Instructor (PSI) [13], and, since 2000, over 4500 PSIs have been trained by Later Life Training (LLT) [personal communication]. PSIs who deliver the intervention have undergone intensive training and assessment that include three face-to-face training days, plus 40 hours of noncontact learning with practical and theoretical assessment. To undertake PSI training, exercise instructors need to hold qualifications in exercise referral and group exercise. PSIs can be employed by the National Health Service (NHS), third sector, local government leisure services or run classes privately.

Participants typically receive between 12 and 24 weeks of FaME free of charge. Where FaME is not funded, the cost to participants varies between £3 and £8 per participant per session. Inclusion criteria vary; however, many services offer FaME to older adults who have fallen in the previous year or display an increased concern about falling. Referral routes include NHS referral (physiotherapy and GP) or self-referral. Often, FaME sessions are run in community venues and leisure centres as ‘rolling programmes’ rather than ‘cohort groups’ to improve programme sustainability. At the end of the 12–24 weeks, there is often onward ‘maintenance’ provision for individuals to transition onto that enables continuation of exercise.

The planning and funding (commissioning) of FaME in England are varied, with no one organisation having statutory responsibility for commissioning fall prevention programmes. In some areas, programmes are commissioned by Integrated Care Boards, which are statutory bodies that commission most NHS provision, alternatively, FaME is funded by local authority public health departments. Fall prevention programmes may also be commissioned by charitable or private sector organisations (e.g. freelance instructors). The funding and landscape of the delivery of FaME remain varied across the UK.

The National Institute for Health and Care Excellence (NICE) has issued guidelines (guideline 161/CG161) [16], currently being updated [17], that offer normative guidance to healthcare professionals (HCPs) and funders on the prevention of falls in older adults. This guidance recommends the prescription of evidence-based strength and balance training for older adults living in the community with a history of recurrent falls and/or balance and gait deficits [16]. The World Falls Guidelines also provide this same guidance [18]. Both guidelines outline that interventions should be individually prescribed, tailored and monitored by an appropriately trained exercise professional. FaME meets current guidance that suggests older adults at risk of falling should receive an effective dose (3+ hours per week) of an exercise intervention that challenges balance to reduce the rate and risk of falls [19]. Despite data supporting the effectiveness of FaME and the intervention meeting current guidelines, FaME is not consistently commissioned across the UK. There remains a paucity of evidence regarding the factors that influence commissioning decisions to adopt, implement and spread evidence-based fall prevention programmes. A recent scoping review has highlighted the need for fall prevention studies to use implementation conceptual frameworks to understand the factors influencing implementation of evidence-based programmes [20]. Adoption in this study is defined as ‘the intention, initial decision, or action to employ an innovation or evidence-based practice’ [21]. Implementation is defined as ‘a change oriented process of putting a plan into action’ [22], and the term spread refers to ‘the adoption and replication, with little modification, of an intervention within a health care system’ [23].

To understand the adoption, implementation and spread of FaME at the community level across three regions of the UK, our study first aimed to identify the factors influencing the decision to commission the programme (adoption) and the factors that were essential to get the programme up and running (implementation). The second aim of the study was to determine the factors and conditions that influenced the spread of the FaME programme within organisations across these areas. Three distinctly different locations, representing a range of geographical characteristics typically seen across the UK [Greater Manchester (a postindustrial conurbation), Leicestershire and Derby in the East Midlands (a mixture of urban and rural areas) and the county of Devon (a rural and coastal area)] were chosen to understand how organisations across the country vary in their adoption, implementation and spread of FaME.

## Methods

### Overview of the study design

Using a qualitative description interpretivist approach [24, 25] semistructured interviews were conducted to gain the perspectives of partners and providers involved in the implementation and delivery of FaME along the community fall prevention pathway [26]. Content analysis of existing documents generated by study organisations was conducted. The documents consisted of meeting notes, policy documentation and reports from organisations within the three UK regions. Further details on the study methodology can be found in the supplementary files (Supplement 1).

### Participant recruitment

Participants were recruited using purposive sampling methods and were approached from a range of occupations to enable the identification of those critical to FaME implementation. Partners with a specific interest in FaME (*n* = 10) included those who were responsible for funding, managing or researching local FaME community fall prevention services. Providers (*n* = 15) included those who had completed the PSI training course delivered by LLT and were currently employed as specialist-trained exercise instructors or HCPs. Participants were asked to provide demographic information (summarised in Table 1) at the end of the interview once the recording had ceased.

**Table 1:** Participant characteristics (*n* = 25)

Characteristics	*N* (%)
*Sex*	
Male	10 (40)
Female	15 (60)
*Professional role*	
Decision-makers/funders	3 (12)
Service leads	7 (28)
Postural Stability Instructors	8 (32)
Clinical healthcare professionals	5 (20)
Academics/researchers	2 (8)
*Employer*	
National Health Service	9 (36)
Community leisure providers	12 (48)
Local government	2 (8)
Academic institutions	2 (8)
*Interview duration* (minutes)	50 ± 12

### Interview topic guide

The semistructured interview topic guides (Supplements 1 and 2) broadly assessed the adoption, implementation and spread of FaME. Partner-specific questions elicited organisational and developmental challenges and successes for FaME adoption and spread. Provider-specific questions elicited further detail about the role of the PSI, alongside organisational programmes of work, networks and connections and service outcomes to address spread.

### Ethical approval

Ethical approval for the study was obtained by the London—City & East Research Ethics Committee reference number 22/PR/0634 and Health Research Authority and Health and Care Research Wales.

### Data collection

Meeting minutes gathered during attendance of meetings with sites that delivered FaME (*n* = 10), policy documentation related to evidence-based fall prevention and organisational reports regarding FaME were provided by sites that took part in the study. Qualitative semistructured interviews were conducted in English by trained qualitative researchers (J.V., F.M., G.B.). Interviews were held in person or over a video conferencing platform depending on the preferences of the participant. Interviews were audio-recorded, transcribed verbatim and then checked for accuracy independently by a study team member. The study reporting follows the Consolidated Criteria for Reporting Qualitative Research checklist [27] (Supplement 3) and the Standards for Reporting Qualitative Research [28] (Supplement 4).

### Data analysis

All study data were analysed using reflexive thematic analysis [29]. For both the interviews (J.V., F.M.) and documents (J.V., A.M.), two separate researchers read the transcripts and documents to familiarise themselves with the data and then inductively coded each transcript and document independently, generating preliminary inductive codes. The preliminary codes for both the interviews and the documents were reviewed by the wider research group at research group meetings and developed into potential themes, alongside agreeing when data saturation had been reached. Codes and themes were then ‘deductively’ mapped and assigned as constructs and domains using the updated 2022 Consolidated Framework for Implementation Research (CFIR) [30]. QSR International’s NVivo 12 software was used for the management of interview data.

## Results

### Domains and constructs

**Figure 1:**
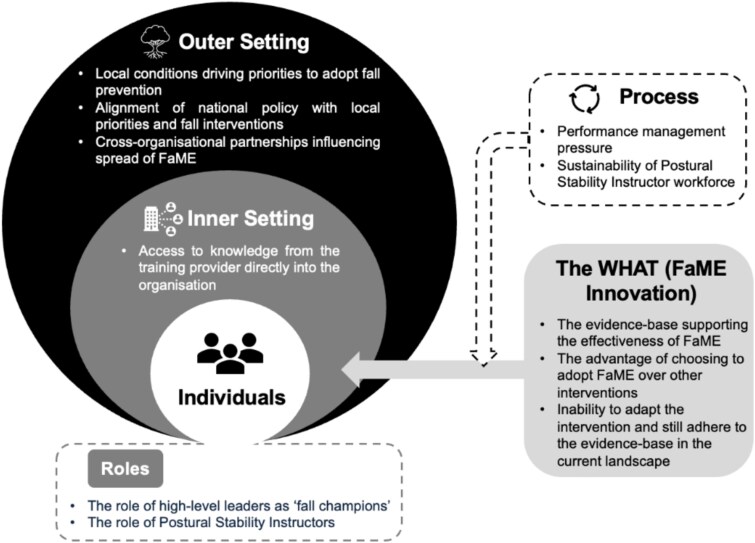
Factors impacting on the adoption and spread of FaME within a community setting. A modified CFIR Figure [30].

Eleven constructs were identified within five broad CFIR domains (‘the innovation’, ‘outer setting’, ‘inner setting’, ‘characteristics of individuals’ and ‘implementation process’). The constructs identified reflect the factors affecting FaME ‘adoption’, ‘implementation’ and ‘spread’ within organisations across the community setting (Figure 1). Constructs related to the ‘adoption’ and ‘implementation’ of FaME were represented across four CFIR domains (‘the innovation’, ‘outer setting’, ‘inner setting’ and ‘characteristics of individuals’). Constructs related to the ‘spread’ of the FaME programme within organisations were also represented across four domains (‘outer setting’, ‘inner setting’, ‘characteristics of individuals’ and ‘implementation process’).

- **The innovation—FaME:** included enablers and challenges regarding the successful but complex nature of the programme.

#### Innovation evidence base—the evidence base supporting the effectiveness of FaME

Partners discussed how decisions to commission fall prevention provision (adoption) were reliant upon evidence demonstrating the effective nature of FaME. Discussions surrounding limited public funding left funders having to prioritise programmes that provided the best return on investment. The research surrounding the ability of FaME to reduce the rate of falls made the selection of the intervention more likely.

*We take a population health management approach, we adopt our clinical effectiveness and our evidence-based research against what we commission, so that has to be really strong. If that’s not there and we don’t have a infinite amount of money, then we need to know that we’re getting the best return on our pound and FaME gives us that evidence. [SH009—Decision-maker and funder].*

#### Innovation relative advantage—the advantage of choosing to adopt FaME over other interventions

FaME was seen to be beneficial in long-term medical cost reduction whilst also having the capability to improve the quality of life of older adults. Partners and providers said that decisions to adopt and recommission the programme were driven by improved participant outcomes (e.g. reduced fall rate). The ability of the programme to positively impact the lives of participants whilst having a positive long-term impact on healthcare costs enabled funders to understand the advantages of adopting and re-commissioning the programme.

*You know when you see first-hand the difference it makes in people’s lives, it’s worth recommissioning compared to other falls interventions. [SH007—Service Lead].*

*We are maximising the funding that we get and as an intervention [FaME], it is certainly really good value for money if you think about the consequences of a fall, in terms of medical costs. [PF007—PSI].*

#### Innovation complexity—tension between adherence to the defined FaME programme and adaptations of it to the local context

Providers of the programme discussed how they struggled with the practicalities of delivering (implementing) the core programme with fidelity in a real-world context. Constraints such as time, funding and instructor capacity often limited full delivery of the programme. Funders often commissioned the programme for a period of 12 weeks, despite the evidence base being 24 weeks. These constraints left decision-makers and funders questioning whether the complex nature of the programme limited the ability to maximise their return on investment.

*Our classes are based on FAME, but they [PSI instructors] have to make judgements about what components of the class to include and not to include due to funding, time constraints and also whether it’s always safe and appropriate to do all the exercises with all the people. In some of the classes, there’s only one person delivering because they may not have a support worker as a secondary person to assist in the classes. They [PSI instructors] work to as much of the evidence base as they can, but sometimes they have to make adaptations and adjustments based on the conditions in front of them and then if we don’t adhere to the evidence-base, are we seeing that return on investment? [SH003—Service Lead].*

- **Outer setting:** included conditions external to the implementing organisations.

Local conditions—local conditions driving priorities to adopt fall prevention

Geographical areas that had larger ageing populations suggested the need for prioritisation of interventions that focused on tackling falls and frailty. Partners and providers suggested that a fast-growing ageing population assisted when determining where to invest public money in a commissioning landscape of competing priorities.

*It’s in my later life strategy plan because frailty and falls is probably one of the biggest needs, especially in [location], because we’ve got I think one of the fastest ageing populations in the country. So, as a later life manager responsible for increasing physical activity and movement in older people it would probably be a bit remiss if it wasn’t in my strategy. [SH005—Service Lead].*

#### Policies and laws—alignment of national policy with local priorities and falls interventions

Partners and funders felt that the adoption and implementation of FaME were largely driven by national policy and published guidelines. The NICE clinical guidelines currently recommend an individualised prescription of strength and balance exercises for older adults at risk of falls. Where fall prevention interventions were able to align and meet these guidelines, the rationale for the adoption of FaME was supported and individuals felt responsible for implementing in accordance with these guidelines. For some organisations, the translation of an intervention that met NICE guidelines, acted as a barrier to adoption. This was because individuals found little available guidance to suggest how to implement evidence-based interventions in a real-world context.

*Yeah. So, I think the priorities within [location] remain the same and the priorities of NHS England align and remain the same. I think we’re very much steered by NHS England and (NHS) Improvement and what they see to be a priority. So what falls out of their long-term plan. I think we’re very much kind of directed by the national vision and what they feel is a priority. [SH009—Decision maker and funder].*

*I mean, everyone talks about how important NICE guidelines are and they absolutely are, but they don’t tell you how to do it. They just say oh, like you need a strength and balance class. . . But that doesn’t help you implement something into practice. [SH001—Academic].*

#### Partnerships and connections—cross-organisational partnerships influencing the spread of FaME:

Many funders, HCPs and PSI instructors were found to be members of local collaboratives across the three geographical areas. Interviewees viewed these partnerships as being conducive to fostering a culture that focused on the importance of fall prevention across the pathway, further supporting the notion that falls are everyone’s business. Partnerships facilitated the spread of the intervention by supporting applications for long-term funding, enabling the longevity of programmes. Others found the collaboratives useful in gaining the attention of key leadership figures, indirectly further supporting access to programme funding.

*You need partnership, leadership buy in and as I say, we’ve had that. We’ve got a good NHS and local government and voluntary sector partnership around falls prevention, so for me that’s key because if you don’t have that. . . you’re not going to get the funding for FaME long term. [PF011—Healthcare Professional].*

- **Inner setting:** included conditions internal to the organisations that are commissioned to deliver FaME.

#### Relational connections—access to knowledge from the training provider directly into the organisation

Organisations found that access to support and knowledge from the training provider facilitated the initial implementation. Early-stage support and specialist knowledge facilitated the set-up of FaME provision and ensured readiness for delivery in organisations that were newly commissioned. In contrast, too much reliance on support from the training provider may present as a barrier to spreading FaME within organisations over time. This is because the support required to set up the service left some managers lacking confidence in the ability to replicate the skills that they had learnt to enable them to set up further FaME provision.

*We now have recruited into an advanced health improvement practitioner post just 8 hours a week, but it’s actually someone who works for [training provider], and so they’re helping to support the set up and they’re an exercise professional by background and so that’s been really helpful to our service. [SH004—Service Lead].*

*We’ve had observations from [name] from [training provider]. So yeah, obviously she is supporting our team quite closely at this time. But yeah, I think that role of having someone who’s obviously very experienced, very knowledgeable in FaME and, you know, falls prevention in general is really key. [PF015—PSI].*

- **Characteristics of individuals:** involved in the commissioning and delivery of FaME that influence adoption and spread of the programme.

### Roles subdomain

#### High-level leaders—The role of high-level leaders as ‘fall champions’

Decision-makers and funders have multiple competing priorities. Organisational managers explained how having a local funder who championed the fall prevention agenda increased the relative priority of funding for FaME. Partners identified the difficulties of accessing resources to implement FaME if local funders did not see fall prevention as a priority. A rapidly changing commissioning landscape in England saw decision-makers and funders frequently changing roles, resulting in the prioritisation of other public health issues. The changing of funders in a local area had a detrimental impact on the continuity of funding and sustainability of fall prevention programmes as a result of the loss of knowledge about the programme.

*Having a good relationship with your commissioner that’s responsible for your fall’s prevention, who also has that passion. I think it’s really important because if the commissioner is not interested, then it’s going to be hard to actually get the resources to be able to implement the programme. [SH003—Service Lead].*

#### Innovation deliverers—the role of Postural Stability Instructors

The success of the programme was attributed to the PSI specialist skillset. Prior to delivering FaME, PSIs receive extensive training in how to tailor and adapt exercise for older adults with multiple comorbidities. This extensive training enhances the PSI skillset that supports the delivery of the programme and the reduction in the rate and risk of falls for their participants. Spreading FaME within an organisation required the PSI to be a source of expertise and have the knowledge of how to set up and run the programme. PSIs are, however, a rate-limiting resource because of their training and expertise, meaning that organisations were unable to increase the number of classes across localities without employing a larger number of PSIs to deliver the classes.

*Our instructors are the only ones that do all of our falls prevention delivery, and we have made that very clear that we realise that they have a different skill set and we have invested in their training so they are the only members of staff that will deliver FaME along the pathway. [PF009—Healthcare Professional].*

*I have now got a member of staff who is assisting me with some of the spread of the programme and she is one of the PSI instructors as well, so I have got knowledge base to draw from when I am asking questions about how things will work. [SH007—Service Lead].*

- **Implementation process:** including the challenges faced by organisations implementing FaME.

#### Reflecting and evaluating—performance management pressure

Funders frequently requested outcome data to evidence the success of the programme. Providers were, therefore, keen to collect outcome data to ensure the recommissioning of programmes. This did, however, present a problem for some providers where PSIs undertook many other roles and so did not have the capacity for collecting performance metrics. PSIs were often only funded for their time whilst delivering FaME, which left time to conduct pre- and postassessments to gather progressional data unfunded and often unobtainable. Overdependence on continuous provision of performance data may limit recommissioning in organisations with limited staff resource. Future funding models are required to incorporate this cost at the outset of delivery.

*With the view that the funding for falls prevention in [Location] is annual usually recurring, but obviously there’s a risk that it won’t be so hopefully we collect enough data to make it. . . to convince everybody that it’s really good value for money, which we know all the evidence suggests it will be. [PF005—PSI].*

*They are not full time PSI instructors; they also have a fitness instructor role as well, so capacity is extremely limited with regards to collecting class data. Maybe once or twice a year this is a possibility [SH004—Service Lead].*

#### Assessing context and needs of innovation deliverers—sustainability of postural stability instructor workforce

The delivery of FaME in all organisations is reliant on a small number of PSIs. The often short-term funding landscape and the uncertainty in continuation of funding have created job insecurity for instructors, resulting in a high staff turnover and a threat to programme continuity. As PSIs are peripatetic and often work alone, organisations that deliver FaME find it difficult to ensure that rural areas are appropriately staffed due to the difficulty in funding PSIs’ travel to remote areas. The paucity of PSIs is a multifactorial issue that spans across job insecurity and the challenges of delivery across large areas as well as lone working. PSIs have actively chosen to retrain into other employment sectors to improve job security. This loss of investment in highly trained exercise instructors may limit the longevity of the programme.

*Some of our PSIs are linked to leisure centres, and they’re on a zero-hour contract. So that’s why we have seen a turnover of staff and some people have moved on to different areas of work and have gone completely outside physical activity and instructing and exercise. So, a massive, massive shame. But they’ve got families to feed and bills to pay. So, it’s understandable. [PF001—PSI].*

*I suppose a couple of things that have hindered spreading the programme is finding enough instructors to cover the whole of the patch, so we’ve tried to put groups on in a couple of localities and it just didn’t work. [PF007—PSI].*

## Discussion

The study investigated the factors affecting the adoption, implementation and spread of the community-based FaME programme across three distinct UK localities. The factors were categorised using the CFIR constructs as ‘the innovation’ (evidence base supporting the effectiveness of FaME, choosing to adopt FaME over other interventions, the inability to adapt the intervention and still adhere to the evidence), ‘outer setting’ (local conditions driving priority, alignment of national policy with local policy, cross organisational partnerships influencing spread), ‘inner setting’ (access to knowledge from the training provider), ‘characteristics of individuals’ (the role of high-level leaders, the role of PSIs) and ‘process’ (performance management pressure, sustainability of PSI workforce). To the authors’ knowledge, this is the first paper to consider the factors affecting FaME implementation using the updated CFIR 2022 framework [30].

Several of the challenges identified for fall prevention implementation are not unique to FaME and have been reported previously [20, 31, 32]; however, the findings in this study indicate that there are a number of important determinants that need to be considered prospectively when implementing FaME. The effective nature of FaME, the intervention’s ability to reduce the rate and risk of falls and a reduction in the associated burden to health and social care services were strong influential factors when choosing what fall prevention intervention to adopt into local practice. Implementing evidence-based interventions with fidelity brings about the best outcomes for recipients [33], and, given the results of several high-quality trials and a ‘real-life’ implementation study [12, 13] demonstrating that FaME improves participant fall outcomes, our results highlight the need to reconsider the FaME implementation processes to ensure that implementation strategies facilitate increased availability of the programme at a national level.

The evidence base for the effectiveness of FaME was an enabler of adoption; however, many providers of the programme struggled with the practicalities of delivering the programme with absolute fidelity in a real-world context. Funding often shortened programme durations because of competing priorities and personal preferences of local decision-makers. Early support during the FaME implementation process could reduce the likelihood of decision-makers shortening and adapting evidence-based interventions based on personal preference and, instead, funding programmes with fidelity that are likely to be most effective. Future work should examine models of delivery and how effective the programme is when core components such as the dose of exercise prescription and the combination of home and supervised sessions deviate in real-world settings. It is proposed that evidence-based fall prevention interventions such as FaME that meet national and world guidelines should form part of a systems approach to fall prevention [16, 18] to bring about the best outcomes for those at greatest risk.

Despite the advantages of implementing FaME as an intervention, context matters when facilitating implementation. The alignment of the intervention with the local needs of an ageing population, national guidelines and policy for fall prevention was a driver for adoption. Current NICE and World Falls Guidelines recommend the prescription of strength and balance exercise for older adults living in the community with a history of recurrent falls and/or balance and gait deficits [16, 18]. The ability of FaME to align with current national and international guidelines supported the rationale for the adoption and spread of the programme, further supporting the suggested integration into routine practice.

The World Guidelines for Falls Prevention and Management for Older Adults highlight that, for successful implementation, regular interaction and engagement with key partners is required [18]. Our study and a recent review [20] found that partnerships and connections significantly facilitated implementation. Engaging and working collaboratively with partners, who had an active enthusiasm for fall prevention, played an influential role in securing funding to adopt the programme. High-level leaders, ‘fall champions’, were seen to be most influential at the adoption stage of implementation. Challenges became present when individuals transitioned out of roles, disrupting the continuity of funding and support for provision. For the facilitation of spread to occur across localities, strong cross-organisational partnerships were identified as being of utmost importance. These findings are in line with previous research which suggests that cross-disciplinary partnerships cannot be neglected due to the multifactorial nature of fall prevention and that multiple partners must be involved along the fall prevention pathway [34, 35].

The present study and others [20, 36] have shown that availability of resources is influential in the delivery of evidence-based interventions. Organisations are required to consider the availability of resources such as appropriate staffing and time to carry out guideline interventions. A lack of available PSIs and high staff turnover were seen to limit spread within organisations. PSIs had limited time to deliver FaME and an even smaller time resource to collect performance data. It may be important for organisations to move beyond numbers and towards an assessment of quality of implementation, supporting the needs of the instructors. For both high-level leaders and instructors delivering FaME, the landscape is ever-changing. The sustainability of the programme is dependent on individuals maintaining roles in fall prevention decision-making and delivery. A model to overcome sustainability is for organisations to offer salaried positions instead of zero-hour contracts. Funding for fall prevention delivery is also required to incorporate budgets to cover regular continuing professional development and attendance at communities of practice to improve peer support for instructors. Future work should specifically examine models of delivery for PSIs to determine factors that contribute to the high turnover of exercise instructor staff.

There are several clear recommendations based on the findings of this study. Fall prevention interventions are required to be evidence-based, meet the needs of older adults in the locality in which they are implemented and meet national/international guidelines. Engagement with appropriate funders and deliverers at the correct stage of the implementation process, alongside forming supportive, collaborative partnerships across the pathway is vital for the continuity of funding and delivery. These findings support recent research to suggest the importance of multilevel and interdisciplinary collaboration in successful fall prevention implementation [37]. Support during the initial adoption phase may be required from a training provider that is able to assist organisations with readiness for delivery. This support will further ensure that organisations have the appropriate skill set to scale up the intervention over time, independently. Process evaluations should take place to ensure successful outcomes are being achieved from the programmes, and we recommend that, where possible, academics with knowledge of evidence-based fall prevention should be part of the implementation of FaME to support ongoing evaluation. This further support and evaluation will highlight successes and areas for improvement in the programmes whilst also addressing the needs of PSIs. A reduction in staff turnover and an appropriate number of PSIs are always required to ensure programme longevity. Lastly, decision-makers and funders need to consider covering payments for collecting performance metric data, alongside travel costs and time for PSIs.

A strength of the present study is that interview participants were recruited purposively at all levels of seniority and roles critical to FaME implementation, from public health decision-makers to HCPs to PSIs across three geographically, socio-demographically and organisationally distinct localities. This approach enabled the investigation of both organisational and operational aspects of FaME implementation and improved the external validity of our results. A further strength is that the recruitment sample was representative of individuals key to the adoption, implementation and delivery of FaME. Individuals were an appropriate mix of professional backgrounds and genders and from varying geographical areas. The use of the CFIR framework further guided the systematic identification of factors influencing the adoption implementation and spread of FaME. This approach assisted in making the data more translatable to other contexts, thus enhancing the generalisability of the results to other fall-prevention intervention contexts. Using both inductive and deductive approaches at different stages of the analysis allowed for the CFIR framework mapping and reporting of how the constructs were reflected in the study setting.

### Study limitations

It may be considered that the study presents limited generalisability through the specific nature of the fall prevention intervention studied (FaME) and the areas of which the data were collected across the UK. It has, however, been outlined how the three regions chosen for data collection were selected based on their distinctive regional characteristics (postindustrial conurbation, urban, rural and coastal areas). The distinctive characteristics of all regions were chosen to represent the landscape of the UK. No factors were determined during the analysis to suggest differences in the enablers and barriers to adoption, implementation and spread based on specific regional localities. Whilst FaME is a stand-alone fall prevention intervention, previous studies have determined several similar barriers and facilitators to the implementation of preventative interventions and the similarities of FaME to other interventions such as Otago have been discussed. We further acknowledge that the findings of this study are susceptible to subjectivity and bias due to the authors’ own perspectives and experiences; however, the use of the CFIR as an analytic framework guided the interpretation of our findings and conclusions to increase the generalisability of the outcomes.

A further limitation is that the CFIR-ERIC (Expert Recommendations for Implementing Change) mapping process developed to support the 2009 iteration of CFIR was used in other fall-prevention implementation studies [38] to enable potential strategies to be used to address barriers identified using CFIR frameworks. The ERIC mapping process is yet to be updated for the 2022 CFIR update, and, whilst it may be possible to make inferences from previous iterations, future work should determine whether strategies to overcome barriers suggested in this paper match those of the ERIC mapping process.

## Conclusion

This study has identified health system challenges and enablers to the adoption, implementation and spread of FaME. The recommendations made in this study are specifically relevant to those tasked with reducing the rate and risk of falls in older adults and to those who fund evidence-based fall prevention programmes such as FaME. For FaME and other fall prevention exercise programmes to be successfully implemented and become sustainable, we recommend providing a toolkit for implementation, continuous funding that enables continuous employment of a large enough trained workforce, supporting instructors to deliver the intervention with fidelity and ongoing evaluation of the effectiveness of the programme. For interventions to be adopted and implemented, the intervention must fit the context and local needs of those receiving the intervention and be supported by influential decision-makers and funders as part of a fall system pathway. The findings from this study will be used to further guide FaME implementation efforts nationally across the UK.

## Supplementary Material

aa-24-2504-File002_afaf083
