# Supplementary material for: Factors influencing fall prevention programmes across three regions of the UK: the challenge of implementing and spreading the Falls Management Exercise (FaME) programme in a complex landscape
Source: Age Ageing. 2025 Apr 10;54(4):afaf083. doi: 10.1093/ageing/afaf083 (PMC11982667; doi:10.1093/ageing/afaf083)
Supplement: aa-24-2504-File002_afaf083 [file aa-24-2504-file002_afaf083.docx]

**Title:** Factors influencing fall prevention programmes across three regions of the UK: The challenge of implementing and spreading the Falls Management Exercise (FaME) programme in a complex landscape.

**Supplement 1:** Additional Methodological Detail

*Participant recruitment*

Organisations that delivered the FaME programme across the three geographical areas submitted expression of interest forms to take part in the study. Upon receiving the expression of interest forms, study researchers contacted individuals from each organisation to determine eligibility and availability to take part in the study. For individuals critical to intervention implementation that were not aligned to a delivering organisation (such as public health decision makers and funders), study researchers contacted individuals via Health Innovation Networks (HINs) (formerly known as Academic Health Science Networks (AHSN)) and via academic institutions. Selection for interview was based on participants knowledge of FaME implementation, operationalisation, and delivery of the programme.

A further 11 participants were approached for interview, alongside the 25 interviewed, but declined due to limited capacity and being unavailable at the time of the study. No repeat interviews were conducted. During interviews a further respondent-driven snowball sampling method was used, and participants were asked to provide details of individuals who may be eligible for interview based on the criteria above. Interview transcripts were returned to participants for acceptance of what was recorded.

*Interview Topic Guide*

Participants were asked to share their experiences of fall prevention service awareness and the effectiveness of commissioned FaME programmes. Partner specific questions were asked to ensure a top-down perspective, whilst further provider specific questions were asked to ensure a bottom-up perspective of adopting, implementing and spreading FaME. Additionally, questions were asked to explore the challenges concerning partnerships along the community fall prevention pathway.

*Data collection*

*Author reflexivity*

The three researchers who conducted the interviews have backgrounds in fall prevention, applied health research and public health. All three researchers had non-medical backgrounds. None of the researchers had any prior engagement or relations with any of the participants before the interviews.

*Data Analysis*

*Selection of CFIR Framework for Analysis*

The CFIR framework was selected as an analytical framework for this study for several reasons. Firstly, the use of CFIR enabled a system-wide evaluation of a multi-level intervention (FaME), within the given context [1]. Secondly, work by Damschroder et al. [2] highlighted that implementation is the critical gateway between an organisational decision to adopt an intervention and the routine use of that intervention. These findings align with the focus of this paper which is to determine the factors affecting the adoption and implementation of FaME and the routine extension of the intervention within local organisations. Lastly, local Health Innovation networks [3] further supported this selection by highlighting the benefits of using implementation science frameworks to examine how theory can present factors about how an intervention can be spread in social systems.

*Domains and Constructs*

Final constructs under the five CFIR domains were refined and agreed based on iterative discussions during wider team meetings. Relevant verbatim quotes were selected to illustrate the main points within each domain and construct. All data were analysed concurrently using the CFIR framework and the findings are presented alongside interview data.

**Supplement 2:** Stakeholder Semi-Structured Interview Topic Guide

**You, and your role**

- Tell me about your organisation and your role within it (prompt: other experience, years in position, passions)
- How does your role relate to others in falls prevention in your area, region, nationally? (prompts: are they influencer, how do they relate to CCGs, councils, charities, government, private providers)

**Implementation and adoption**

- Tell me about what is available in your area/service in terms of falls prevention exercise programmes (prompts: is it based on a particular evidence-based programme; how was this decided)?
- How did this particular service come about? (prompt: has it always been the same; how has it evolved/changed over time; who has been involved; when did it start; why did it start)
- What sorts of things have contributed to the service development and how has it helped?  (prompt: people; organisations; services; policy; guidelines; relationships; toolkit)
- What sort of things have hindered progress? (Prompt: things that didn’t happen or go as planned; unforeseen circumstances)? What impact did this have?
- How do you think service users view the programmes and the ways they are run? Why?
- Is there anything about your service you would change? If yes: can you please describe in what circumstances you might do this? And why?
- What could others learn from your service and how it has developed in order to develop their own service

**Does the programme work?**

- How effective do you feel your programme/service is? (Prompt: how do you evaluate)?
- Is there anything you would do differently to enable more/different people to take part (prompt: health inequalities)
- What is stopping you offering the programme/service to all those at risk of falling in your area? What could be done to overcome these issues?

**Closing remarks**

- Anything else you would like to share with me
- Are there key people you think we should approach to interview?

**Supplement 3:** Providers Semi-Structured Interview Topic Guide

**You, and your role**

- Tell me about your organisation and or role in it, background in falls prevention or related area? (prompt: other experience, years in position, passions)
- **For PSIs** tell me how long you have been a PSI and about the organisation/s you have provided FaME instruction for and your role within it (prompt: providing for public or voluntary sector; experience with different providers)

**Implementation and adoption**

- Tell me about your local falls exercise programme/service (prompt: is it based on a particular evidence based programme; when was it set up; are there core components that you include or not; how decided, COVID)
- Can you tell me about the pathways into falls exercise provision in your area? (prompt: criteria, referrers, signposting, follow on classes, other opportunities, key links)
- Talk me through any assessments you carry out as part of the programme? (prompt: timings; challenges)
- Can you tell me about your approach to FaME and your PSI classes (prompt: what a typical class is like/entails, progression, hybrid?)
- How do you support people during and outside your classes (prompt: any strategies; behaviour change; homework; written exercises; peer support; phone calls, diary, social)
- How do you monitor quality of the programmes, and your own fidelity & continuous improvement (prompt: observations, discussion groups, feedback, reviews, CoP)

**Does the programme work**

- How effective do you feel your programme/service is? (Prompt: how do you evaluate, service user views)?
- What has worked well in delivering FaME? Have you adapted it at all (prompt: what, why, how, impact)
- What have been the challenges so far? Have you found ways to overcome them; Do you see different challenges moving forwards?

**For Service Providers only:**

- Can you describe procurement/tendering/commissioning process (prompt: Why did you bid/competitors, reasons for success).
- What did and didn’t work implementing/delivering FaME in your area? (prompt: observations, who and why were influential, any modifications, did you need to negotiate changes)
- What are your plans to continue FaME or a version of it in the future? Why? How will you do this? (prompt: funding, models, etc)

**Closing remarks**

- Anything else you would like to share with me
- Are there key people you think we should approach to interview

**Supplement 4:** Consolidated criteria for reporting qualitative studies (COREQ) checklist.

| **No** | **Item** | **Guide questions/description** | **Performed by** |
| --- | --- | --- | --- |
| **Domain 1: Research team and reflexivity** | | | |
| **Personal Characteristics** | | | |
| 1. | Interviewer/facilitator | Which author/s conducted the interview or focus group? | Trained qualitative researchers (JV, FM, and GB) (methodology section) |
| 2. | Credentials | What were the researcher's credentials? *E.g. PhD, MD* | JV PhD  FM PhD  GB MPH  AM PhD |
| 3. | Occupation | What was their occupation at the time of the study? | JV – Research Associate  FM – Research Fellow/Lecturer  GB – Assistant Professor/Acting Consultant Public Health  AM – Research Fellow |
| 4. | Gender | Was the researcher male or female? | Females |
| 5. | Experience and training | What experience or training did the researcher have? | Training and experience in qualitative research methods-including moderating focus groups, coding, and thematic analysis |
| **Relationship with participants** | | | |
| 6. | Relationship established | Was a relationship established prior to study commencement? | No |
| 7. | Participant knowledge of the interviewer | What did the participants know about the researcher? e*.g. personal goals, reasons for doing the research* | The participants knew the reason for the researcher conducting the research - i.e. to find factors that influenced implementation of FaME |
| 8. | Interviewer characteristics | What characteristics were reported about the interviewer/facilitator? e.g. *Bias, assumptions, reasons and interests in the research topic* | Reasons for interest in the research topic and background in research/academia |
| **Domain 2: study design** | | | |
| **Theoretical framework** | | | |
| 9. | Methodological orientation and Theory | What methodological orientation was stated to underpin the study? *e.g. grounded theory, discourse analysis, ethnography, phenomenology, content analysis* | Thematic analysis framework proposed by Braun and Clarke, followed by mapping themes and codes to the Consolidated Framework for Implementation Research (CFIR) |
| **Participant selection** | | | |
| 10. | Sampling | How were participants selected? *e.g. purposive, convenience, consecutive, snowball* | Purposive sampling |
| 11. | Method of approach | How were participants approached? e*.g. face-to-face, telephone, mail, email* | Via email |
| 12. | Sample size | How many participants were in the study? | 25 participants engaged in the semi-structured interview process |
| 13. | Non-participation | How many people refused to participate or dropped out? Reasons? | 11 participants declined invitation (primary reason- not available at the time of discussion, limited capacity to complete the interview) |
| **Setting** | | | |
| 14. | Setting of data collection | Where was the data collected? e*.g. home, clinic, workplace* | Interviewer and interviewee sat in private rooms; interviews conducted using Microsoft Teams |
| 15. | Presence of non-participants | Was anyone else present besides the participants and researchers? | No |
| 16. | Description of sample | What are the important characteristics of the sample? *e.g. demographic data, date* | Reported in methods section |
| **Data collection** | | | |
| 17. | Interview guide | Were questions, prompts, guides provided by the authors? Was it pilot tested? | Topic guide was created by authors and informed using implementation framework theory |
| 18. | Repeat interviews | Were repeat interviews carried out? If yes, how many? | No |
| 19. | Audio/visual recording | Did the research use audio or visual recording to collect the data? | Audio recordings with in-built MS Teams transcription |
| 20. | Field notes | Were field notes made during and/or after the interview or focus group? | Yes |
| 21. | Duration | What was the duration of the interviews or focus group? | Semi-structured interviews durations ranged between 44-64 minutes |
| 22. | Data saturation | Was data saturation discussed? | Yes |
| 23. | Transcripts returned | Were transcripts returned to participants for comment and/or correction? | Yes |
| **Domain 3: analysis and findings** | | | |
| **Data analysis** | | | |
| 24. | Number of data coders | How many data coders coded the data? | 3 |
| 25. | Description of the coding tree | Did authors provide a description of the coding tree? | Yes |
| 26. | Derivation of themes | Were themes identified in advance or derived from the data? | Derived from data and deductively coded to the CFIR |
| 27. | Software | What software, if applicable, was used to manage the data? | QSR NVivo 12 |
| 28. | Participant checking | Did participants provide feedback on the findings? | Anonymised data was discussed with PPIE group members to gain feedback |
| **Reporting** | | | |
| 29. | Quotations presented | Were participant quotations presented to illustrate the themes / findings? Was each quotation identified? e*.g. participant number* | Yes, the quotes are presented with participant number |
| 30. | Data and findings consistent | Was there consistency between the data presented and the findings? | Yes |
| 31. | Clarity of major themes | Were major themes clearly presented in the findings? | Yes |
| 32. | Clarity of minor themes | Is there a description of diverse cases or discussion of minor themes? | Yes |

**Supplement 5:** Standards for Reporting Qualitative Research (SRQR).

| **Title and abstract** | | Page |
| --- | --- | --- |
|  | **Title** - Concise description of the nature and topic of the study Identifying the study as qualitative or indicating the approach (e.g., ethnography, grounded theory) or data collection methods (e.g., interview, focus group) is recommended | 1 |
|  | **Abstract** - Summary of key elements of the study using the abstract format of the intended publication; typically includes background, purpose, methods, results, and conclusions | 1 |
|  |  |  |
| **Introduction** | |  |
|  | **Problem formulation** - Description and significance of the problem/phenomenon studied; review of relevant theory and empirical work; problem statement | 4 |
|  | **Purpose or research questio**n - Purpose of the study and specific objectives or questions | 4 |
|  |  |  |
| **Methods** | |  |
|  | **Qualitative approach and research paradigm** - Qualitative approach (e.g., ethnography, grounded theory, case study, phenomenology, narrative research) and guiding theory if appropriate; identifying the research paradigm (e.g., postpositivist, constructivist/ interpretivist) is also recommended; rationale** | 4 |
|  | **Researcher characteristics and reflexivity** - Researchers’ characteristics that may influence the research, including personal attributes, qualifications/experience, relationship with participants, assumptions, and/or presuppositions; potential or actual interaction between researchers’ characteristics and the research questions, approach, methods, results, and/or transferability | Supplementary file 1 |
|  | **Context** - Setting/site and salient contextual factors; rationale** | 5 |
|  | **Sampling strategy** - How and why research participants, documents, or events were selected; criteria for deciding when no further sampling was necessary (e.g., sampling saturation); rationale** | 5 and Supplementary file 1 |
|  | **Ethical issues pertaining to human subjects** - Documentation of approval by an appropriate ethics review board and participant consent, or explanation for lack thereof; other confidentiality and data security issues | 5 |
|  | **Data collection methods** - Types of data collected; details of data collection procedures including (as appropriate) start and stop dates of data collection and analysis, iterative process, triangulation of sources/methods, and modification of procedures in response to evolving study findings; rationale** | 5, 6 |
|  | **Data collection instruments and technologies** - Description of instruments (e.g., interview guides, questionnaires) and devices (e.g., audio recorders) used for data collection; if/how the instrument(s) changed over the course of the study | 5, 6 |
|  | **Units of study** - Number and relevant characteristics of participants, documents, or events included in the study; level of participation (could be reported in results) | 5, 6 |
|  | **Data processing** - Methods for processing data prior to and during analysis, including transcription, data entry, data management and security, verification of data integrity, data coding, and anonymization/de-identification of excerpts | 6 |
|  | **Data analysis** - Process by which inferences, themes, etc., were identified and developed, including the researchers involved in data analysis; usually references a specific paradigm or approach; rationale** | 6 |
|  | **Techniques to enhance trustworthiness** - Techniques to enhance trustworthiness and credibility of data analysis (e.g., member checking, audit trail, triangulation); rationale** | 6 and Supplementary file 1 |
|  |  |  |
| **Results/findings** | |  |
|  | **Synthesis and interpretation** - Main findings (e.g., interpretations, inferences, and themes); might include development of a theory or model, or integration with prior research or theory | 7, 8, 9, 10, 11, 12 |
|  | **Links to empirical data** - Evidence (e.g., quotes, field notes, text excerpts, photographs) to substantiate analytic findings | 7, 8, 9, 10, 11, 12 |
|  |  |  |
| **Discussion** | |  |
|  | **Integration with prior work, implications, transferability, and contribution(s) to the field -** Short summary of main findings; explanation of how findings and conclusions connect to, support, elaborate on, or challenge conclusions of earlier scholarship; discussion of scope of application/generalizability; identification of unique contribution(s) to scholarship in a discipline or field | 13, 14, 15, 16, 17 |
|  | **Limitations** - Trustworthiness and limitations of findings | 16, 17 |
|  |  |  |
| **Other** | |  |
|  | **Conflicts of interest** - Potential sources of influence or perceived influence on study conduct and conclusions; how these were managed | 18 |
|  | **Funding** - Sources of funding and other support; role of funders in data collection, interpretation, and reporting | 18 |
|  |  |  |
|  | *The authors created the SRQR by searching the literature to identify guidelines, reporting standards, and critical appraisal criteria for qualitative research; reviewing the reference lists of retrieved sources; and contacting experts to gain feedback. The SRQR aims to improve the transparency of all aspects of qualitative research by providing clear standards for reporting qualitative research. |  |
|  |  |  |
|  | **The rationale should briefly discuss the justification for choosing that theory, approach, method, or technique rather than other options available, the assumptions and limitations implicit in those choices, and how those choices influence study conclusions and transferability. As appropriate, the rationale for several items might be discussed together. |  |

**References**

1. Keith RE, Crosson JC, O’Malley AS, et al.; Using the Consolidated Framework for Implementation Research (CFIR) to produce actionable findings: a rapid-cycle evaluation approach to improving implementation. *Implementation Science* 2017;**12**:1-12.

2. Damschroder LJ, Aron DC, Keith RE, et al.; Fostering implementation of health services research findings into practice: a consolidated framework for advancing implementation science. *Implementation science* 2009;**4**:1-15.

3. Manchester HI; Using Implementation Science to support adoption and spread. 2021.
